# 2-{Hy­droxy[1-(4-meth­oxy­phen­yl)-4-oxo-3-phenyl­azetidin-2-yl]meth­yl}acrylonitrile

**DOI:** 10.1107/S1600536811032247

**Published:** 2011-08-17

**Authors:** C. M. Sai Prasanna, K. Sethusankar, R. Rajesh, R. Raghunathan

**Affiliations:** a Department of Physics Ethiraj College for Women, Chennai 600 008, India; b Department of Physics RKM Vivekananda College (Autonomous), Chennai 600 004, India; c Department of Organic Chemistry University of Madras Guindy Campus, Chennai 600 025, India

## Abstract

In the title compound, C_20_H_18_N_2_O_3_, the β-lactam ring is essentially planar, having a maximum deviation of 0.0291 (15) Å for the N atom, and perpendicular to the phenyl ring [dihedral angle = 85.55 (11)°]. The carbonitrile side chain is almost linear, the C—C—N angle being 176.8 (2)°. The crystal packing is stabilized by inter­molecular O—H⋯O and C—H⋯O inter­actions.

## Related literature

For uses of acrylonitrile derivatives, see: Ambrosi *et al.* (1994 ▶). For the pharmacological properties of β-lactam derivatives, see: Brakhage (1998 ▶). For related structures, see: Sundaresan *et al.* (2008 ▶); Kamala *et al.* (2008 ▶). For related geometrical parameters, see: Nizam Mohideen *et al.* (2007 ▶).
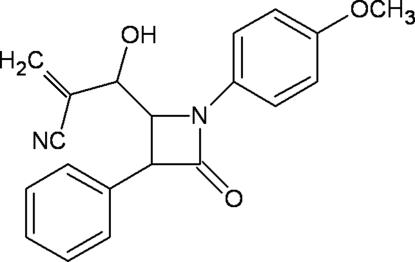


## Experimental

### 

#### Crystal data

C_20_H_18_N_2_O_3_
*M* *_r_* = 334.36
Monoclinic, 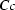

*a* = 9.9694 (3) Å
*b* = 19.8196 (6) Å
*c* = 9.6013 (3) Å
β = 112.718 (1)°
*V* = 1749.93 (9) Å^3^
*Z* = 4
Mo *K*α radiation
μ = 0.09 mm^−1^
*T* = 295 K
0.30 × 0.20 × 0.20 mm

#### Data collection

Bruker Kappa APEXII CCD diffractometer
10596 measured reflections
4368 independent reflections
3621 reflections with *I* > 2σ(*I*)
*R*_int_ = 0.019

#### Refinement

*R*[*F* ^2^ > 2σ(*F* ^2^)] = 0.038
*wR*(*F* ^2^) = 0.100
*S* = 1.01
4368 reflections
228 parameters
2 restraints
H-atom parameters constrained
Δρ_max_ = 0.16 e Å^−3^
Δρ_min_ = −0.15 e Å^−3^

### 

Data collection: *APEX2* (Bruker, 2004 ▶); cell refinement: *SAINT* (Bruker, 2004 ▶); data reduction: *SAINT*; program(s) used to solve structure: *SHELXS97* (Sheldrick, 2008 ▶); program(s) used to refine structure: *SHELXL97* (Sheldrick, 2008 ▶); molecular graphics: *ORTEP-3* (Farrugia, 1997 ▶); software used to prepare material for publication: *SHELXL97* and *PLATON* (Spek, 2009 ▶).

## Supplementary Material

Crystal structure: contains datablock(s) global, I. DOI: 10.1107/S1600536811032247/rk2289sup1.cif

Structure factors: contains datablock(s) I. DOI: 10.1107/S1600536811032247/rk2289Isup2.hkl

Supplementary material file. DOI: 10.1107/S1600536811032247/rk2289Isup3.cml

Additional supplementary materials:  crystallographic information; 3D view; checkCIF report

## Figures and Tables

**Table 1:** Hydrogen-bond geometry (Å, °)

*D*—H⋯*A*	*D*—H	H⋯*A*	*D*⋯*A*	*D*—H⋯*A*
O3—H3⋯O1^i^	0.82	1.90	2.716 (2)	172
C9—H9⋯O2^ii^	0.98	2.50	3.467 (2)	169

Symmetry codes: (i) 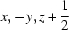; (ii) 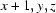.

**Fig. 1.**
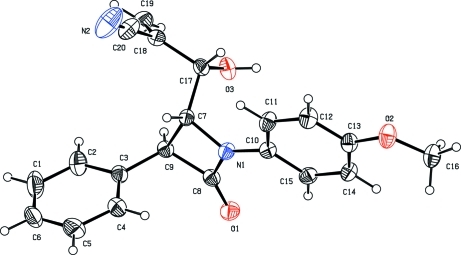
The molecular structure of the title compound with the atom numbering scheme. Displacement ellipsoids are drawn at 30% probability level. H atoms are presented as a small spheres of arbitrary radius.

**Fig. 2.**
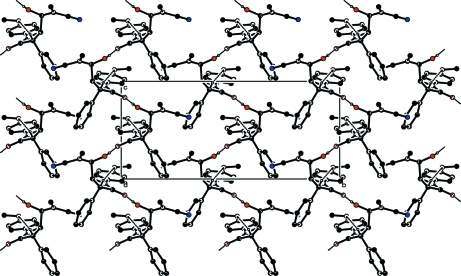
Packing arrangement of the title compound in the unit cell as viewed down the a axis. The O–H···O and C–H···O intermolecular interactions indicated by dashed lines.

## supplementary crystallographic information

### Comment

Acrylonitriles are useful intermediates in organic synthesis and are capable of undergoing many useful organic transformations (Ambrosi *et al.*, 1994), for example, into pyrazole, isoxazole and pyrimidine derivatives. β-Lactams are one of the best known and most extensively studied class of compounds due to their biological activities. The most commonly used β-lactam antibiotics for the therapy of infectious diseases are penicillin and cephalosporin (Brakhage, 1998). X-ray analysis confirms the molecular structure and atom connectivity as illustrated in Fig. 1.

In the title compound C_20_H_18_N_2_O_3_, due to conjugation in the C19═C18–C20≡N2 moiety, the bond length between C18 and C20 (1.429 (3)Å) shows a significant shortening (Nizam Mohideen *et al.*, 2007). The β-lactam ring is essentially planar and the keto O1 atom deviates from this mean plane by -0.1095 (13)°. The methoxy phenyl (C11-C15) and the phenyl rings (C1-C6) form dihedral angles of 15.66 (10)° and 85.55 (11)°, respectively, with the β-lactam ring. The sum of the angles around N1 atom of the β-lactam ring system accounts for 359.5 (3)° which is in accordance with *sp*^2^ hybridization. The phenyl ring and methoxy phenyl ring are *cis* related with respect to the β-lactam ring.

The bond angles at C18 deviate significantly from regular trigonal geometry. The bond angle around C20, in the chains of atoms C18/C20/N2, is 176.8 (2)° and thus the carbonitrile side chain is almost linear. The torsion angle of -112.87 (19)° for C7–C17–C18═C19 indicates a deviation of the β-lactam ring from the plane of the olefinic double bond. The methoxy group is slightly twisted from the plane of the phenyl ring (C10-C15) to which it is attached as evidenced by the torsion angle C14-C13-O2-C16 of 7.8 (2)°. The interplanar angle between the phenyl (C1-C6) and methoxy phenyl group (C10-C15) is 81.04 (9)°. The title compound exhibits structural similarities with the already reported related structures (Sundaresan *et al.*, 2008, Kamala *et al.*, 2008).

The crystal packing is stabilized by O–H···O and C–H···O hydrogen bonds *via* O3–H3···O1^i^ and C9–H9···O2^ii^ intermolecular interactions, viewed down the *a* axis. Symmetry codes: (i) *x*, *-y*, *z*+1/2; (ii) *x*+1, *y*, *z*. The packing view of the title compound is shown in Fig. 2.

### Experimental

To the reaction mixture of 1-(4-methoxyphenyl)-4-oxo-3-phenylazetidine-2-carbaldehyde (1 mmol) with acrylonitrile (2 mmol), a catalytic quantity of 1,4-diazabicyclo[2.2.2]octane (10-15 mol %) was added. The reaction mixture was left standing at room temperature in a stoppered sample flask. The progress of the reaction was monitored by *TLC* over a period of several days. After a period of 10 days, the *TLC* revealed the presence of a product. The reaction mixture was dissolved in ethyl acetate and washed with aqueous HCl solution (0.25 *M*) and water followed by brine solution. The organic layer was separated and dried over sodium sulfate. Filtering and evaporation of the organic solvent was done under reduced pressure. The product was separated by flash column chromatography using hexane and ethyl acetate as an eluent (1:9) to give colourless solid. The product was dissolved in chloroform and heated for two minutes. The resulting solution was subjected to crystallization by slow evaporation of the solvent resulting in single crystals suitable for XRD studies.

### Refinement

The hydrogen atoms were placed in calculated positions with C–H = 0.93Å to 0.98Å, O–H = 0.82Å and refined in the riding model with fixed isotropic displacement parameters: *U*_iso_(H) = 1.2*U*_eq_(C) for aromatic, methylene, methine groups, *U*_iso_(H) = 1.5*U*_eq_(C) for methyl group and *U*_iso_(H) = 1.5*U*_eq_(O) for hydroxy groups.

The 1876 Friedel pairs were merged ('MERG 2' instruction in *SHELXL* refinement).

### Crystal data

**Table:**

C_20_H_18_N_2_O_3_	*F*(000) = 704
*M**_r_* = 334.36	*D*_x_ = 1.269 Mg m^−^^3^
Monoclinic, *C**c*	Mo *K*α radiation, λ = 0.71073 Å
Hall symbol: C -2yc	Cell parameters from 4368 reflections
*a* = 9.9694 (3) Å	θ = 2.4–29.7°
*b* = 19.8196 (6) Å	µ = 0.09 mm^−^^1^
*c* = 9.6013 (3) Å	*T* = 295 K
β = 112.718 (1)°	Block, colourless
*V* = 1749.93 (9) Å^3^	0.30 × 0.20 × 0.20 mm
*Z* = 4	

### Data collection

**Table:**

Bruker Kappa APEXII CCD diffractometer	3621 reflections with *I* > 2σ(*I*)
Radiation source: fine-focus sealed tube	*R*_int_ = 0.019
graphite	θ_max_ = 29.7°, θ_min_ = 2.4°
ω and φ scans	*h* = −13→13
10596 measured reflections	*k* = −25→27
4368 independent reflections	*l* = −11→13

### Refinement

**Table:**

Refinement on *F*^2^	Primary atom site location: structure-invariant direct methods
Least-squares matrix: full	Secondary atom site location: difference Fourier map
*R*[*F*^2^ > 2σ(*F*^2^)] = 0.038	Hydrogen site location: inferred from neighbouring sites
*wR*(*F*^2^) = 0.100	H-atom parameters constrained
*S* = 1.01	*w* = 1/[σ^2^(*F*_o_^2^) + (0.0577*P*)^2^ + 0.1723*P*] where *P* = (*F*_o_^2^ + 2*F*_c_^2^)/3
4368 reflections	(Δ/σ)_max_ < 0.001
228 parameters	Δρ_max_ = 0.16 e Å^−^^3^
2 restraints	Δρ_min_ = −0.15 e Å^−^^3^

### Special details

**Table:**

Geometry. All s.u.'s (except the s.u. in the dihedral angle between two l.s. planes) are estimated using the full covariance matrix. The cell s.u.'s are taken into account individually in the estimation of s.u.'s in distances, angles and torsion angles; correlations between s.u.'s in cell parameters are only used when they are defined by crystal symmetry. An approximate (isotropic) treatment of cell s.u.'s is used for estimating s.u.'s involving l.s. planes.
Refinement. Refinement of *F*^2^ against ALL reflections. The weighted *R*-factor *wR* and goodness of fit *S* are based on *F*^2^, conventional *R*-factors *R* are based on *F*, with *F* set to zero for negative *F*^2^. The threshold expression of *F*^2^ > σ(*F*^2^) is used only for calculating *R*-factors(gt) *etc*. and is not relevant to the choice of reflections for refinement. *R*-factors based on *F*^2^ are statistically about twice as large as those based on *F*, and *R*-factors based on ALL data will be even larger.

### Fractional atomic coordinates and isotropic or equivalent isotropic displacement parameters (Å^2^)

**Table:**

	*x*	*y*	*z*	*U*_iso_*/*U*_eq_	
C1	0.6009 (3)	0.19499 (12)	0.1527 (3)	0.0809 (7)	
H1	0.6877	0.2116	0.1515	0.097*	
C2	0.6025 (2)	0.15799 (11)	0.2755 (3)	0.0630 (5)	
H2	0.6902	0.1499	0.3558	0.076*	
C3	0.47523 (17)	0.13317 (8)	0.27943 (17)	0.0401 (3)	
C4	0.3470 (2)	0.14539 (10)	0.1573 (2)	0.0579 (5)	
H4	0.2599	0.1284	0.1570	0.069*	
C5	0.3470 (3)	0.18264 (12)	0.0352 (2)	0.0779 (7)	
H5	0.2600	0.1908	−0.0461	0.093*	
C6	0.4739 (3)	0.20732 (11)	0.0336 (3)	0.0746 (7)	
H6	0.4737	0.2324	−0.0482	0.089*	
C7	0.38921 (15)	0.13134 (7)	0.50213 (17)	0.0340 (3)	
H7	0.3452	0.1742	0.4561	0.041*	
C8	0.36425 (16)	0.03948 (7)	0.39009 (16)	0.0379 (3)	
C9	0.47470 (16)	0.09626 (7)	0.41628 (17)	0.0362 (3)	
H9	0.5727	0.0827	0.4844	0.043*	
C10	0.16463 (15)	0.06120 (7)	0.49049 (16)	0.0359 (3)	
C11	0.10281 (17)	0.11362 (8)	0.5408 (2)	0.0443 (4)	
H11	0.1396	0.1571	0.5466	0.053*	
C12	−0.01237 (18)	0.10169 (8)	0.5822 (2)	0.0471 (4)	
H12	−0.0528	0.1371	0.6165	0.057*	
C13	−0.06861 (16)	0.03725 (8)	0.57319 (18)	0.0414 (3)	
C14	−0.00915 (17)	−0.01503 (8)	0.51980 (19)	0.0425 (4)	
H14	−0.0471	−0.0584	0.5123	0.051*	
C15	0.10649 (17)	−0.00288 (8)	0.47766 (18)	0.0410 (3)	
H15	0.1452	−0.0379	0.4406	0.049*	
C16	−0.2330 (2)	−0.03522 (11)	0.6291 (3)	0.0599 (5)	
H16A	−0.2710	−0.0556	0.5308	0.090*	
H16B	−0.3083	−0.0327	0.6680	0.090*	
H16C	−0.1540	−0.0620	0.6957	0.090*	
C17	0.47215 (16)	0.13644 (8)	0.67345 (17)	0.0391 (3)	
H17	0.4046	0.1492	0.7208	0.047*	
C18	0.58853 (18)	0.19031 (8)	0.70558 (18)	0.0433 (4)	
C19	0.7295 (2)	0.17793 (11)	0.7631 (2)	0.0607 (5)	
H19A	0.7953	0.2131	0.7778	0.073*	
H19B	0.7629	0.1341	0.7890	0.073*	
C20	0.5348 (2)	0.25736 (10)	0.6650 (3)	0.0602 (5)	
N1	0.28665 (13)	0.07467 (6)	0.45591 (15)	0.0381 (3)	
N2	0.4860 (3)	0.30955 (10)	0.6326 (4)	0.1008 (8)	
O1	0.34670 (14)	−0.01631 (6)	0.33461 (15)	0.0505 (3)	
O2	−0.18217 (13)	0.03094 (6)	0.61854 (16)	0.0557 (3)	
O3	0.53772 (13)	0.07510 (6)	0.73290 (14)	0.0525 (3)	
H3	0.4856	0.0542	0.7661	0.079*	

### Atomic displacement parameters (Å^2^)

**Table:**

	*U*^11^	*U*^22^	*U*^33^	*U*^12^	*U*^13^	*U*^23^
C1	0.0945 (18)	0.0779 (15)	0.098 (2)	−0.0164 (13)	0.0673 (17)	0.0082 (13)
C2	0.0580 (11)	0.0681 (11)	0.0739 (13)	−0.0036 (9)	0.0375 (10)	0.0104 (10)
C3	0.0488 (8)	0.0364 (7)	0.0431 (8)	0.0012 (6)	0.0268 (7)	−0.0027 (6)
C4	0.0618 (10)	0.0669 (11)	0.0434 (10)	−0.0110 (9)	0.0186 (8)	0.0037 (8)
C5	0.1023 (18)	0.0790 (15)	0.0425 (11)	−0.0110 (13)	0.0172 (11)	0.0081 (10)
C6	0.129 (2)	0.0563 (12)	0.0573 (12)	−0.0166 (12)	0.0570 (14)	−0.0006 (9)
C7	0.0361 (7)	0.0303 (6)	0.0398 (7)	−0.0006 (5)	0.0192 (6)	−0.0018 (5)
C8	0.0429 (8)	0.0365 (7)	0.0377 (8)	0.0052 (6)	0.0193 (7)	0.0000 (6)
C9	0.0379 (7)	0.0364 (7)	0.0381 (7)	0.0036 (5)	0.0186 (6)	−0.0004 (5)
C10	0.0334 (7)	0.0358 (7)	0.0390 (7)	0.0036 (6)	0.0146 (6)	−0.0007 (6)
C11	0.0438 (8)	0.0321 (7)	0.0628 (11)	0.0002 (6)	0.0271 (7)	−0.0025 (7)
C12	0.0438 (8)	0.0399 (8)	0.0655 (11)	0.0089 (7)	0.0298 (8)	0.0003 (7)
C13	0.0322 (7)	0.0448 (8)	0.0474 (9)	0.0027 (6)	0.0155 (6)	0.0056 (6)
C14	0.0415 (8)	0.0352 (8)	0.0521 (9)	−0.0046 (6)	0.0195 (7)	−0.0016 (6)
C15	0.0432 (8)	0.0359 (7)	0.0442 (8)	0.0009 (6)	0.0171 (7)	−0.0056 (6)
C16	0.0520 (10)	0.0602 (11)	0.0747 (14)	−0.0111 (8)	0.0324 (10)	0.0041 (9)
C17	0.0426 (8)	0.0425 (8)	0.0381 (8)	−0.0011 (6)	0.0221 (6)	−0.0006 (6)
C18	0.0497 (9)	0.0449 (9)	0.0386 (8)	−0.0053 (7)	0.0206 (7)	−0.0064 (6)
C19	0.0509 (10)	0.0615 (11)	0.0645 (12)	−0.0068 (9)	0.0166 (9)	−0.0034 (9)
C20	0.0520 (10)	0.0496 (11)	0.0780 (13)	−0.0107 (8)	0.0239 (9)	−0.0131 (9)
N1	0.0395 (6)	0.0323 (6)	0.0473 (7)	−0.0010 (5)	0.0221 (5)	−0.0062 (5)
N2	0.0825 (14)	0.0460 (11)	0.161 (3)	−0.0015 (9)	0.0328 (14)	−0.0037 (12)
O1	0.0618 (7)	0.0393 (6)	0.0584 (7)	−0.0014 (5)	0.0321 (6)	−0.0114 (5)
O2	0.0462 (7)	0.0517 (7)	0.0801 (9)	0.0010 (5)	0.0364 (6)	0.0068 (6)
O3	0.0558 (7)	0.0535 (7)	0.0536 (7)	0.0034 (5)	0.0271 (6)	0.0171 (5)

### Geometric parameters (Å, °)

**Table:**

C1—C6	1.361 (4)	C11—C12	1.373 (2)
C1—C2	1.383 (3)	C11—H11	0.9300
C1—H1	0.9300	C12—C13	1.384 (2)
C2—C3	1.375 (2)	C12—H12	0.9300
C2—H2	0.9300	C13—O2	1.367 (2)
C3—C4	1.382 (2)	C13—C14	1.387 (2)
C3—C9	1.506 (2)	C14—C15	1.382 (2)
C4—C5	1.385 (3)	C14—H14	0.9300
C4—H4	0.9300	C15—H15	0.9300
C5—C6	1.361 (4)	C16—O2	1.423 (2)
C5—H5	0.9300	C16—H16A	0.9600
C6—H6	0.9300	C16—H16B	0.9600
C7—N1	1.4675 (18)	C16—H16C	0.9600
C7—C17	1.533 (2)	C17—O3	1.3938 (19)
C7—C9	1.559 (2)	C17—C18	1.518 (2)
C7—H7	0.9800	C17—H17	0.9800
C8—O1	1.2105 (18)	C18—C19	1.319 (3)
C8—N1	1.3645 (19)	C18—C20	1.429 (3)
C8—C9	1.526 (2)	C19—H19A	0.9300
C9—H9	0.9800	C19—H19B	0.9300
C10—C15	1.382 (2)	C20—N2	1.134 (3)
C10—C11	1.387 (2)	O3—H3	0.8200
C10—N1	1.4047 (19)		
			
C6—C1—C2	120.8 (2)	C10—C11—H11	119.8
C6—C1—H1	119.6	C11—C12—C13	120.30 (14)
C2—C1—H1	119.6	C11—C12—H12	119.8
C3—C2—C1	120.4 (2)	C13—C12—H12	119.8
C3—C2—H2	119.8	O2—C13—C12	115.46 (14)
C1—C2—H2	119.8	O2—C13—C14	125.06 (14)
C2—C3—C4	118.34 (16)	C12—C13—C14	119.48 (14)
C2—C3—C9	120.81 (15)	C15—C14—C13	120.13 (14)
C4—C3—C9	120.79 (14)	C15—C14—H14	119.9
C3—C4—C5	120.60 (19)	C13—C14—H14	119.9
C3—C4—H4	119.7	C10—C15—C14	120.17 (14)
C5—C4—H4	119.7	C10—C15—H15	119.9
C6—C5—C4	120.3 (2)	C14—C15—H15	119.9
C6—C5—H5	119.8	O2—C16—H16A	109.5
C4—C5—H5	119.8	O2—C16—H16B	109.5
C1—C6—C5	119.6 (2)	H16A—C16—H16B	109.5
C1—C6—H6	120.2	O2—C16—H16C	109.5
C5—C6—H6	120.2	H16A—C16—H16C	109.5
N1—C7—C17	113.52 (12)	H16B—C16—H16C	109.5
N1—C7—C9	87.60 (10)	O3—C17—C18	109.23 (12)
C17—C7—C9	114.72 (12)	O3—C17—C7	110.82 (13)
N1—C7—H7	112.9	C18—C17—C7	108.52 (12)
C17—C7—H7	112.9	O3—C17—H17	109.4
C9—C7—H7	112.9	C18—C17—H17	109.4
O1—C8—N1	131.25 (14)	C7—C17—H17	109.4
O1—C8—C9	135.95 (14)	C19—C18—C20	120.90 (16)
N1—C8—C9	92.80 (11)	C19—C18—C17	124.13 (16)
C3—C9—C8	117.38 (13)	C20—C18—C17	114.94 (15)
C3—C9—C7	115.56 (12)	C18—C19—H19A	120.0
C8—C9—C7	84.78 (11)	C18—C19—H19B	120.0
C3—C9—H9	112.2	H19A—C19—H19B	120.0
C8—C9—H9	112.2	N2—C20—C18	176.8 (2)
C7—C9—H9	112.2	C8—N1—C10	135.24 (12)
C15—C10—C11	119.48 (14)	C8—N1—C7	94.46 (11)
C15—C10—N1	121.69 (13)	C10—N1—C7	129.77 (11)
C11—C10—N1	118.83 (13)	C13—O2—C16	117.93 (14)
C12—C11—C10	120.40 (14)	C17—O3—H3	109.5
C12—C11—H11	119.8		
			
C6—C1—C2—C3	−0.2 (4)	C12—C13—C14—C15	0.7 (2)
C1—C2—C3—C4	0.9 (3)	C11—C10—C15—C14	−2.3 (2)
C1—C2—C3—C9	−176.48 (18)	N1—C10—C15—C14	176.76 (14)
C2—C3—C4—C5	−1.0 (3)	C13—C14—C15—C10	0.9 (2)
C9—C3—C4—C5	176.35 (18)	N1—C7—C17—O3	52.33 (16)
C3—C4—C5—C6	0.5 (3)	C9—C7—C17—O3	−46.30 (16)
C2—C1—C6—C5	−0.3 (4)	N1—C7—C17—C18	172.26 (12)
C4—C5—C6—C1	0.2 (4)	C9—C7—C17—C18	73.64 (15)
C2—C3—C9—C8	−146.86 (16)	O3—C17—C18—C19	8.0 (2)
C4—C3—C9—C8	35.9 (2)	C7—C17—C18—C19	−112.87 (19)
C2—C3—C9—C7	115.42 (18)	O3—C17—C18—C20	−173.48 (15)
C4—C3—C9—C7	−61.8 (2)	C7—C17—C18—C20	65.61 (19)
O1—C8—C9—C3	68.9 (2)	O1—C8—N1—C10	2.8 (3)
N1—C8—C9—C3	−111.64 (14)	C9—C8—N1—C10	−176.66 (16)
O1—C8—C9—C7	−174.95 (18)	O1—C8—N1—C7	174.71 (17)
N1—C8—C9—C7	4.50 (11)	C9—C8—N1—C7	−4.78 (12)
N1—C7—C9—C3	113.74 (13)	C15—C10—N1—C8	9.6 (3)
C17—C7—C9—C3	−131.40 (14)	C11—C10—N1—C8	−171.31 (17)
N1—C7—C9—C8	−4.18 (11)	C15—C10—N1—C7	−159.80 (15)
C17—C7—C9—C8	110.68 (13)	C11—C10—N1—C7	19.2 (2)
C15—C10—C11—C12	2.1 (2)	C17—C7—N1—C8	−111.32 (13)
N1—C10—C11—C12	−177.01 (15)	C9—C7—N1—C8	4.67 (12)
C10—C11—C12—C13	−0.4 (3)	C17—C7—N1—C10	61.25 (19)
C11—C12—C13—O2	179.40 (16)	C9—C7—N1—C10	177.24 (14)
C11—C12—C13—C14	−1.0 (3)	C12—C13—O2—C16	−172.56 (17)
O2—C13—C14—C15	−179.66 (15)	C14—C13—O2—C16	7.8 (2)

### Hydrogen-bond geometry (Å, °)

**Table:**

*D*—H···*A*	*D*—H	H···*A*	*D*···*A*	*D*—H···*A*
O3—H3···O1^i^	0.82	1.90	2.716 (2)	172
C9—H9···O2^ii^	0.98	2.50	3.467 (2)	169

Symmetry codes: (i) *x*, −*y*, *z*+1/2; (ii) *x*+1, *y*, *z*.

## References

[bb1] Ambrosi, H.-D., Duczek, W., Ranm, M., Gründemann, E., Schulz, B. & Jahnisch, K. (1994). *Liebigs Ann. Chem.* pp. 1013–1018.

[bb2] Brakhage, A. A. (1998). *Microbiol. Mol. Biol. Rev.* **62**, 547-585. 10.1128/mmbr.62.3.547-585.1998 PMC98925 9729600

[bb3] Bruker (2004). *APEX2* and *SAINT* Bruker AXS Inc., Madison, Wisconsin, USA.

[bb4] Farrugia, L. J. (1997). *J. Appl. Cryst.* **30**, 565.

[bb5] Kamala, E. T. S., Nirmala, S., Sudha, L., Arumugam, N. & Raghunathan, R. (2008). *Acta Cryst.* E**64**, o887–o888. 10.1107/S1600536808010428 PMC2961124 21202371

[bb6] Nizam Mohideen, M., Kannan, P. S., Subbiah Pandi, A., Ramesh, E. & Raghunathan, R. (2007). *Acta Cryst.* E**63**, o4756.

[bb7] Sheldrick, G. M. (2008). *Acta Cryst.* A**64**, 112–122. 10.1107/S0108767307043930 18156677

[bb8] Spek, A. L. (2009). *Acta Cryst.* D**65**, 148–155. 10.1107/S090744490804362X PMC2631630 19171970

[bb9] Sundaresan, S., Ramesh, P., Arumugam, N., Raghunathan, R. & Ponnuswamy, M. N. (2008). *Acta Cryst.* E**64**, o2042. 10.1107/S1600536808030961 PMC2959299 21201234

